# On Typhoid Fever, a Paper Read to the Cook Co. Medical Society

**Published:** 1852-12

**Authors:** Erial McArthur


THE NORTH-WESTERN
MEDICAL AND SURGICAL JOURNAL.

NEW SERIES.

VOL. I. DECEMBER, 1852. No. 8.

### ORIGINAL COMMUNICATIONS.

ARTICLE I.—*On Typhoid Fever, a Paper read to the Cook Co. Medical Society, by* Erial McArthur, M. D.

Writers and teachers of medicine have generally divided fever into Idiopathic and Symptomatic—the former depending upon no local cause, while the latter depends upon some local irritation.

Some writers have, for practical considerations, given the characteristic divisions of Dynamic and Adynamic Fevers. Our attention, however, is called to no general view of fever at this time.

Typhoid Fever is Idiopathic or "Essential Fever" in its character; and in its distinctive form is Adynamic. The predisponent cause of this fever is a subject about which there has been much dispute. I shall define it as being a specific principle or poison, emanating from vegetable or animal decompositions, and sometimes from both combined. This poison is received into the system in various degrees of concentration and combinations diluted in atmospheric air, through the medium of the lungs, the skin, and the prima via. It commingles with the blood, and thus produces its effects upon the nervous system.

Chemistry has thus far failed to define definitely what this latent principle or poison is, or even fully to detect it in form or in substance. And yet the almost universal consent of mankind places the certainty of it beyond a cavil or a doubt. I shall for the present assume that this miasma or poison produces alike the different grades or types of fever known under the familiar names of Agues, Intermittents, Remittents, Continued (including Typhoid and Typhus), Yellow Fevers, &c. It will not be necessary for my purpose at this time, to notice the arguments usually resorted to for the purpose of proving the positions which have been assumed, or to examine the "Cryptogamous origin" of fever, which has been beautifully and fancifully set forth in the *American Journal of the Medical Sciences*, by Professor Mitchell, of Philadelphia, or any other theory extant for the causation of fever, but to proceed at once to our subject.

Typhoid Fever, it may be said, is caused by miasm arising from decayed and decaying vegetable or animal substances, and in many instances from both,—it being absorbed and conveyed to the sanguiferous system, producing, as a secondary result, prostration and derangement of the nervous forces. It is from this derangement that the morbid changes are going forward, which are commonly spoken of as the premonitory symptoms. The blood becomes changed in some of its vital elements and combinations, and the want of the usual nervous stimulation to the lungs and circulatory system in general, results in a want of the proper arterialization of the blood and a consequent increase of general debility. As there is a *decrease* of the vital air, so there is a relative *increase* of carbon in the circulating fluids of the body; and hence, in some extreme cases, we have apnœa and coma, produced severally by different states and conditions of the blood, as well as by different variations of force in its circulation. These conditions, it is true, may be produced by engorgement of different viscera of the body, caused by mechanical derangement only. As this disease advances, the fluids accumulate in dependant parts of the body to a greater or less extent according to the decrease of the vital forces. In some severe cases the lungs become hepatized and infiltrated with pus, and the bronchial tubes are clogged with viscid and frothy mucus.

Sudden affliction, continued watching, constant depressing emotions, arising from care, anxiety, fear, and other affections of the mind—may and sometimes do serve as exciting causes to develop this disease. It is so generally diffused through society at times, that there appears to be an epidemic influence prevailing as an efficient and exciting cause. By a large number of medical writers it is said to be even contagious, and consequently received by coming in contact with those affected with it. This position seems hardly conclusive, when the history of the disease is fairly understood, and especially if we do not take into our calculation its development in large Hospitals.

### IMMEDIATE CAUSE OF DEATH.

This to a great extent may depend upon debility of the heart, or in other words, a continuous relaxation of its muscles. And yet death from asthenia alone, seldom obtains, it being more generally combined with other causes. It has been found by autopsical examinations of subjects who have died from this disease, that there are more traces of organic lesions and derangements in the abdominal viscera than in the thorax and head.

### PATHOLOGY.

Peyer's glands are found enlarged in some subjects; in some cases they are of a reddish cast; in others they are of a grayish color and dotted with black points. The follicles in some subjects are found ulcerated — parts having sloughed off — leaving irregular and ragged ulcers with thickened edges. In some instances the follicles are pale and level, in others puffed up with fungous growth of different variations of color. These glands being situated beneath the villous coat in the lower portion of the ileum, it may be in some instances more difficult to diagnose their diseased condition while the patient is living, than if they were in a higher portion of the intestines. Ulceration, however, is more fully developed here, it being seldom formed in the jejunum or duodenum, while inflammation of a different kind is often developed in these latter divisions. Hence the mesenteric glands in their locality become hard, swollen, and tender. These, however, become so inflamed subsequently, as a sequence of the inflammation and ulceration of the mucous glands. These alterations of structure in general, fully account for the variety of symptoms developed in the severer forms of Typhoid Fever. Thus we have diarrhœa and hæmorrhage in the latter stages of the disease; also pain and uneasiness in the abdominal region. The redness, thickening, ulceration, and sloughing are more generally observed when the disease becomes epidemic or general. The violence of the symptoms does not always depend, however, upon the degree of inflammation and ulceration, for in many instances, when the disease is most violent, these circumstances are mostly or entirely wanting. The ulceration of the bowels sometimes heals, and parts thus become sound and healthy, again performing their usual functions; at other times the ulceration continues until the bowel is perforated and its contents pass into the abdominal cavity. The spleen is more frequently found diseased and changed in its structure than any other glandular viscus of the abdomen in the severer forms of Typhoid Fever. It occasionally becomes dark colored, soft, apparently decayed and even rotten. This may be considered presumptive evidence of the malarious origin of the disease. There is necessarily no organic derangements or lesions of the brain or thorax in this disease, although it may become complicated by some local affection of cerebral or thoracic origin.

### VARIETIES.

Although this disease may be considered specific in its character, there are variations in form depending upon climate, season of the year, and epidemic and other influences. It generally commences with arterial excitement, and sometimes of a high grade, though more frequently with the form of the inflammatory fever denominated by Cullen and other writers as Synochus. Hence, when this disease commences with high arterial excitement and a general sthenic condition of the system, its tendency is soon apparent, as it often passes, and that sometimes rapidly, to a lingering feebleness of the vital powers.

The history of this disease shows plainly that since Asiatic Cholera has prevailed in Europe and in this country, its character is more of an asthenic form than it was previous to this time. This fact is worthy of especial notice by the Profession, as it corresponds with the history of disease in general. Indeed, it has become an axiom in the annals of our science, that radical changes are continually going on in the character and form of almost every variety and distinction of disease. The importance, therefore, of medical men carefully and assiduously investigating these continued changes, and adapting their treatment thereto, cannot be too strenuously advocated and insisted upon, and, especially by writers and teachers of medicine. This disease sometimes appears as an Epidemic, at other times as an Endemic, and more frequently still in this country as Sporadic, being in many instances, as it were, engrafted on to other varieties of disease. It is characterised at times as being accompanied with a peculiar rash, and at other times this circumstance may be entirely wanting and ulceration and inflammation of the bowels obtain. Its time of duration may be for a few days only, or it may continue for many weeks. In the winter season it is likely to be complicated with local affections of the throat and lungs; in the summer by extreme debility and depression; and in the autumn by Diarrhœa, Dysentery, &c.

SYMPTOMS.

This disease is peculiar in respect to the great variety of symptoms which are more or less manifested in every patient afflicted with it. In cases attended with considerable inflammatory action in the incipient stages, there is pain in the back and limbs accompanied with trembling of the lower extremities, headache, vertigo, flushed face, &c. There is loss of appetite, the tongue is coated with a whitish fur, eyes are red and swollen, the bowels are costive in some, while in others diarrhœa obtains. Where there is absence of this high febrile condition not unfrequently there is a drooping sensation, a shivering fit, the patient is easily tired, in some the countenance is pale and languid, in others a redness of countenance, owing to capillary congestion, is manifested, the patient has a confusion of ideas, an abstracted expression of the eyes, is reluctant to exercise, has evil forebodings of the future, a fatuitous and fixed indifference, an inexpressive apathy to surrounding objects, want of appetite, has a torpid inactive state of the bowels. With some the urine is pale, but more frequently it is of a dark color and scanty, and there is a settled dizziness accompanied with febrile oppression. There is a brown coating of the tongue, in some it is even black, and as the disease advances the tongue becomes parched, dry and sensitive to the touch. In severe cases the tongue becomes smooth and glossy and of a reddened aspect, and there is great difficulty in protruding it. Nausea and vomiting are occasional symptoms, the matter ejected being green or yellow, and much of it bile. Abdominal tenderness is found in a large share of patients and frequently tympanites, and especially is there hardness and tenderness over the cæcal region. A slight eruption affects many patients with Typhoid Fever, it is however for the most part confined to the regions of the bowels and to the lower extremities. As the disease advances sordes accumulate upon the teeth. Deafness is of frequent occurrence, and sometimes from the incipiency of the disease.—There is a general tendency to sleep. Coma occurs in some cases. The patient becomes more and more indifferent to surrounding objects and actions, and as the disease progresses is apparently little concerned in the issue of his case. Some patients with Typhoid Fever have Diarrhœa at the commencement with fœtid and dark colored stools. The disease is sometimes complicated with Bronchitis and Pneumonia. As the Fever continues to advance the patient's intelligence generally diminishes, there is in some cases continued delirium, the patient is more and more reluctant to voluntary movements, sinks gradually lower in the bed, is unwilling or incapable of turning himself on the side, the knees are drawn up, the voice becomes feeble and hollow, has difficult deglutition, lies with his mouth open, has subsultus tendinum, a tremor of the tongue and acuteness of hearing. And again with others in this stage of the disease, the sense of sight, touch, smell and hearing become more or less impaired, although, perhaps, there is no special complaint in relation to either, and it is only found out by accidental circumstances. There is sometimes retention of urine, and at other times involuntary and continued dribbling of it. In the epidemic form of this fever, there are rosy blotches scattered over different parts of the body, somewhat raised above the general surface of the skin; they may be partially diminished under pressure though not fully so. This rash differs from that affection of the skin called petechiæ, and yet the two forms may in some measure commingle together. Hæmorrhage also takes place at this stage. This may occur from lesions of the bowels from ulceration, or it may be of a strictly passive kind, arising from putridity or laxity of the tissues and a morbid condition of the fluids. An offensive and peculiar fœtor exhales from the skin, the tongue becomes black, dry and fissured, sordes continue to accumulate upon the teeth, portions of the body most pressed upon by long confinement begin to slough away, and death finally closes the scene.

DIAGNOSIS.

The diseases most likely to be confounded with, or taken for Typhoid Fever, and from which it is most difficult to distinguish it, are Typhus and Remittent Fevers and diseases of the brain. It will not be so difficult as heretofore to determine the distinction between these diseases, in consequence of the light thrown upon the subject by analytical investigations during the last fifty years. Although essentially alike in some general features, there appear to be distinctive characters and symptoms. Young persons are more frequently attacked with Typhoid Fever, and those who have passed the meridian of life are more subject to Typhus. The premonitory period is of longer duration in the former disease than in the latter, the fever is more of the sthenic type, and it more frequently prevails in the autumnal season. Diarrhœa is not only more frequent in Typhoid Fever than in Typhus, but it is more active, requiring prompt and vigorous treatment to arrest its progress, whereas in Typhus it is less frequently present and more easily checked. The congestion of the capillaries of the surface is more marked and distinct in the former disease; and when delirium affects the patient, it assumes more of an active form at the commencement, while it is of a passive form in Typhus Fever. Cephalalgia is a prominent symptom in Typhoid Fever, and for the most part is entirely wanting in the Typhus. In the former disease, deafness more or less marked, vascular injection of the conjunctiva, a reddish tongue and vomiting are frequently observed as characteristic of that kind of fever; but in Typhus Fever these symptoms are seldom present, and when observed are very slight comparatively. Sordes on the teeth are sooner developed in Typhus, and are also more troublesome and continue longer than in Typhoid. Hæmorrhage from the nose and bowels is somewhat common in Typhoid, and is generally wanting in Typhus Fever. Tenderness on pressure is a common symptom in the hypogastrium and iliac regions in the former fever, and peritonitis, either from perforation of the bowels or from some other cause, sometimes occurs in this form of fever, though generally absent in Typhus fever. Eruptions of the skin are more frequent in Typhus and of a dark red color, not being elevated from the surrounding surface, while in Typhoid Fever, the eruption when it is present, is of a rose color and elevated. In Typhus Fever there is more cough and pneumonic complication than in Typhoid. There is a strong dissimilarity in the two diseases in this respect. The pulse is stronger in Typhoid and more frequent in the Typhus type. There is much more general nervous prostration in the latter disease than in the former.

In distinguishing this disease from Remittent Fever, we observe in the latter more of a tendency to periodicity, for although this fever may be somewhat continuous, yet there appears to be a manifest difference in the violence of the fever at different times of the day. There is much less tendency to general nervous prostration and to deafness in Remittent Fever. In Remittent Fever there is more nausea and vomiting of bile, more constant thirst, more tenderness in the epigastrium and at the same time less in the iliac regions, less, if any, sordes accumulated upon the teeth, little or no flatulency and meteorism, and less diarrhœa. In Remittent Fever there is generally no eruption of the skin, and very seldom Epistaxis, while in Typhoid Fever the eruption and hæmorrhage are somewhat frequent. There is often a yellow tinge of the skin and conjunctiva in the former disease, but this seldom occurs in the Typhoid Fever. Remittent Fever attacks all ranks and conditions of men, and of every period of life from the prattling infant to the octogenarian, and in general prevails only in malarious districts, and for the most part ends in Intermittent Fever. Typhoid Fever, however, generally attacks young persons, and prevails at times when, and places where other diseases are not so prevalent.

From diseases of the brain, Typhoid Fever may be distinguished by its greater tendency to Diarrhœa, Dysentery, &c. While in cerebral disease there is a general liability to constipation, a greater sensibility to light and somnolency. There is likewise at times, in this last-mentioned disease, a vacant stare and peculiar expression of the eyes, which, when once seen, will always be recognized subsequently, and which seldom, if ever, occurs in any other class of diseases.

From most other diseases Typhoid Fever will be found to differ in so many appreciable and diversified symptoms as to make the diagnosis easy and at the same time conclusive.

### TREATMENT.

In regard to treatment I shall be confined to my own views and convictions, founded upon some little experience and reflection, and that without any special regard to what may be deemed authority, neither compiling from it on the one hand, nor purposely shunning its precepts on the other. I shall endeavor to be brief, only giving some general principles as applicable thereto.

Typhoid Fever doubtless has a tendency to an intrinsic limitation, varying from several days to as many weeks. There is much danger from the adaptation of treatment "*to the name of the disease*"—rather than properly adapting it to the symptoms manifesting derangement of the general powers of life according to acknowledged principles of therapeutical science. Certain geographical locations have a tendency to give type in some measure to the disease which prevails, whether epidemic, sporadic, or otherwise. In the New England States, and the eastern part of the Middle States, for instance, there is a manifest liability to complications with diseases affecting the throat and lungs. In the Western and Southern States, and the western part of the Middle States, on the other hand, a tendency to bilious derangement prevails. In these different localities, consequently, an appreciable modification of treatment would be required to meet the exigencies and wants of the patient in Typhoid Fever as well as any other disease. It is therefore essential to the successful treatment of this disease, that these various modifications arising from locality and differences of climate be borne in mind.

We have previously stated that since the prevalence of Asiatic Cholera, there has been an appreciable modification of Typhoid Fever. This disease has now a greater tendency to nervous prostration.—Ataxial symptoms are sooner developed and take deeper hold of the system than previous to the advent of this pestilential scourge of humanity. In cases of Typhoid Fever attended with high arterial excitement in the first stage of the disease—general and local blood-letting might be in some patients indicated, though only at the outset of the course. Its tendency to pass rapidly from this condition of excitement to a low and nervous prostration of the vital powers, admonishes us to do at an early period whatever we do in the way of depleting or reducing the system. In this climate, where the disease for the most part is complicated with an excess, or want of the proper secretion of the bile, a vitiated or loaded condition of the stomach and bowels—mild emetics and laxatives may be given at first with good effect, in a large proportion of cases. Thus, if the tongue is covered with a brown coating, and there is a general derangement of the digestive organs, as in my experience proves often to be the case, x. to xv. grains of Calomel, with a like quantity of Ipecac., given, will have the effect, first, to relieve the stomach of this bilious matter, and by following it in three or four hours, if necessary, with Castor Oil or a Seidlitz Powder, the bowels will be gently evacuated and the patient relieved of vitiated ingesta, and be in a better condition for the reception of tonics and opiates. If the tongue should be coated with dark brown fur along the centre, and especially towards its root, with a reddened and dry condition of its edges and tip, accompanied with more or less bilious Diarrhœa—Calomel combined with Opium or Sulph. Morphia, would doubtless relieve the bowels of vitiated and irritating excretions, and at the same time have the effect to correct the secretions of the liver and of the prima via. In a goodly number of cases of this disease under my care, I have found that Sulph. Quinia seemingly tended to shorten the duration of this fever, although in many instances my expectations were disappointed.

After the use of the calomel and ipecac., or in some patients the pill hydrarg., ℞ Sulph. quinia grs. xv. to ℈j., Sulph. morphia gr. j., mix, and divide into four powders. Take one, and repeat every three to four hours until all are taken. When there is much determination of blood to the head, quinia is not indicated; and its effects are more uniformly beneficial where a laxative has first been given. I sometimes give Dover's powder in combination with the quinine rather than the morphia. If there is but a moderate degree of pain in the head, or if there is a little indication of a lax condition of the bowels, I prefer the latter combination.

If the quinia and anodyne combined fail to check the progress of the fever, sulph. morphia and tart. emetic, given in small quantities and at proper intervals, may be found beneficial. A cold solution of sup. tart. potassæ has the effect to cool the feverish condition of the system which at times prevails.

Opium, or in some instances the sulph. morphia, in cases attended with diarrhœa, has the effect usually to check its progress. Where dysenteric symptoms obtain, the opium, sulph. morphia, or Dover's powder, singly or in combination with sugar of lead, has often proved sufficient to arrest the irritation and mucous discharges. Enema of mucilage and opium, in case the last-named remedies fail, will generally prove sufficient to check it, either singly or in combination.

Ablutions with cold or tepid water, judiciously attended to according to the circumstances and condition of the patient, having reference at the sametime to the progress of the disease, are very useful.

The tinct. or ext. of valerian, castor, and opiate anodynes, may be relied upon with a good degree of confidence, to palliate general nervous irritability. It is sometimes found necessary to give Port wine, brandy, or carb. ammonia, to hold up and sustain the forces of the system, against the general prostration and sinking, which in many cases prevails. In patients laboring under a complication of a bronchial character, the usual expectorants, such as tart. emetic, ipecac., Vir. snake root, squills, &c., are useful to allay and even to check the progress of the bronchial affection. These may be given alone, or in combination with opiates or stimulants, as the circumstances may seem to require.

From some experience of the beneficial effects of the Cod-liver oil, I hesitate not to place it in the list of remedial agents in the treatment of Typhoid Fever, and especially when there is complication with disease of the lungs and mesenteric glands. In cases attended with wasting discharges and sinking of the powers of life, this remedy will be found, I think, useful to meet the indication; and, if the stomach will bear the cautious use of it in small quantities, its beneficial effect will make good any reasonable expectation.

Very much depends upon the use of a proper diet, and that to be varied somewhat, and carefully adapted to the condition and wants of the patient in the different stages of the disease.

